# The ethnicity and gut microbiota hypothesis: analyzing multifactorial interactions and their health implications

**DOI:** 10.3389/fmicb.2026.1765775

**Published:** 2026-02-19

**Authors:** Lei Liang, Xuxiang Zhang, Xin Nian

**Affiliations:** 1 Department of Endocrinology, The First Affiliated Hospital of Kunming Medical University, Kunming, Yunnan, China; 2 Department of Endocrinology, Anhui Provincial Hospital, The First Affiliated Hospital, University of Science and Technology of China, Hefei, Anhui, China

**Keywords:** circadian rhythm, diet, elevation, ethnicity, genes, geography, gut microbiota, intestinal flora

## Abstract

This manuscript investigates the impact of ethnic differences on gut microbiota, with a focus on multifactorial interactions and associated health implications. It defines the “Ethnicity-Gut Microbiota” phenomenon, wherein distinct ethnic groups exhibit significant variations in the composition, structure, and function of gut microbiota. We searched for relevant references in the Web of Science and PubMed databases using keywords up to December 1, 2025. Three key influencing factors are examined: genetic factors such as TLR and FUT2 gene polymorphisms that affect microbial colonization, geographical factors including elevation, soil, circadian rhythm, and temperature that alter microbial diversity, and lifestyle factors such as diet, physical activity, sleep, and health-related pharmaceutical use that shape microbial profiles. These factors interact dynamically and mutually influence one another, ultimately determining the gut microbiota differences across various ethnic groups. This research holds significant value for understanding disease disparities, optimizing drug administration, guiding public health strategies, and investigating human evolution. Future research directions include large-scale multiethnic studies, multi-omics integration, and exploration of microbial functional differences. Ultimately, this work aims to advance precision health initiatives for diverse ethnic populations.

## 1 The concept of “Ethnicity-Gut Microbiota”

The human gut harbors a complex and diverse microbial ecosystem, housing approximately 3600 bacterial species and 10^13^−10^14^ bacterial cells (Liu et al., 2020; Lloyd-Price et al., 2016; Leviatan et al., 2022). This gut microbial community carries 124 times more genes than the human nuclear genome, earning it the title of the “second human genome” (Qin et al., 2010).

The gut microbiota constitutes an extremely complex, dynamic ecosystem whose host relationship has long transcended simple parasitism or symbiosis. The scientific community now widely recognizes it as a holobiont: the host gut furnishes a warm, moist, nutrient-rich, and relatively stable niche for microbial survival, while the microbiota aids the host in food digestion, essential nutrient synthesis, immune maturation, pathogen defense, and intestinal barrier modulation.

Once thought to develop in a sterile uterine environment, the fetus instead undergoes extensive microbial colonization during birth. This microbiota then matures progressively over the following 3 years, approaching an adult-like profile. Vaginally delivered neonates first encounter maternal vaginal and birth canal microbiota; accordingly, their initial microbial communities are dominated by *Lactobacillus, Prevotella*, and other taxa that promote early immune development. In contrast, neonates born via cesarean section are first exposed to maternal skin and environmental microbes, resulting in initial gut microbiota that closely resemble skin flora, with *Staphylococcus* and *Corynebacterium* as key constituents.

Feeding mode is the primary driver of microbial succession. Human milk is rich in human milk oligosaccharides (HMOs), which are indigestible by infants but act as prebiotics to selectively nourish specific taxa—particularly *Bifidobacterium*. Consequently, breastfed infants exhibit extremely high intestinal *Bifidobacterium* abundance, creating an environment that suppresses pathogenic growth. In contrast, formula milk lacks HMOs and contains more readily utilizable nutrients, resulting in relatively greater microbial diversity, reduced *Bifidobacterium* levels, and elevated *Bacteroides* and *Clostridium* abundances.

Solid food introduction triggers substantial shifts in gut microbiota structure. The expanding complexity of dietary components (e.g., plant polysaccharides, proteins) provides substrates for a more diverse microbial community, driving a transition from a *Bifidobacterium*-dominated “infant-type” microbiota to an “adult-type” profile enriched in Bacteroidetes and Firmicutes. By ~3 years of age, gut microbiota structure and function typically stabilize, approximating adult levels.

Notably, interindividual variability in gut microbiota composition persists among adults with similar delivery methods and feeding patterns, with such taxonomic divergence being particularly pronounced across distinct ethnic cohort (Byrd et al., 2020).

For instance, a comparative study of healthy individuals in Belgium and Japan revealed marked disparities in gut microbiota composition between the two populations (Ishikawa et al., 2013). Similarly, a Malaysian study focusing on four distinct ethnic groups—Malays, Chinese, Indians, and Jakun—residing within the same community confirmed notable differences in their gut microbiota, underscoring the strong association between ethnicity and gut microbiota composition (Dwiyanto et al., 2021b).

Our research team previously conducted a comparative study on gut microbiota by recruiting healthy Han and Dai individuals in Yunnan Province, China. After rigorously controlling for confounding factors such as age, gender, disease status, medication use, and environmental conditions, we still detected significant differences in the composition and structure of gut microbiota between the two ethnic groups (Liang et al., 2023b). Additionally, we found that the gut microbiota composition and structure of the healthy Dai population in our study bore a resemblance to that of the Thai population studied by Lee et al. (2017). Given that the Dai people of Yunnan Province, China, and the Thai people—the dominant ethnic group in Thailand—share a common ethnic origin, we put forward the “Ethnicity-Gut Microbiota Hypothesis.”

This review systematically elucidates the concept of the “Ethnicity-Gut Microbiota Hypothesis” and its influencing factors, discussing its implications for current research in clinical studies, public health, and anthropology. Finally, we explore future research directions by addressing existing gaps and limitations in the field of the “Ethnicity-Gut Microbiota” research.

We searched the Web of Science and PubMed databases using keywords such as “Ethnicity,” “Race,” “Gut Microbiota,” “Intestinal flora,” “Genes,” “Geography,” “Lifestyle,” “Elevation,” “Soil,” “Circadian Rhythm,” “Temperature,” “Diet,” “Sports,” “Sleep,” and “Pharmaceuticals.” The cutoff date for this search was December 1, 2025.

**Figure 1:**
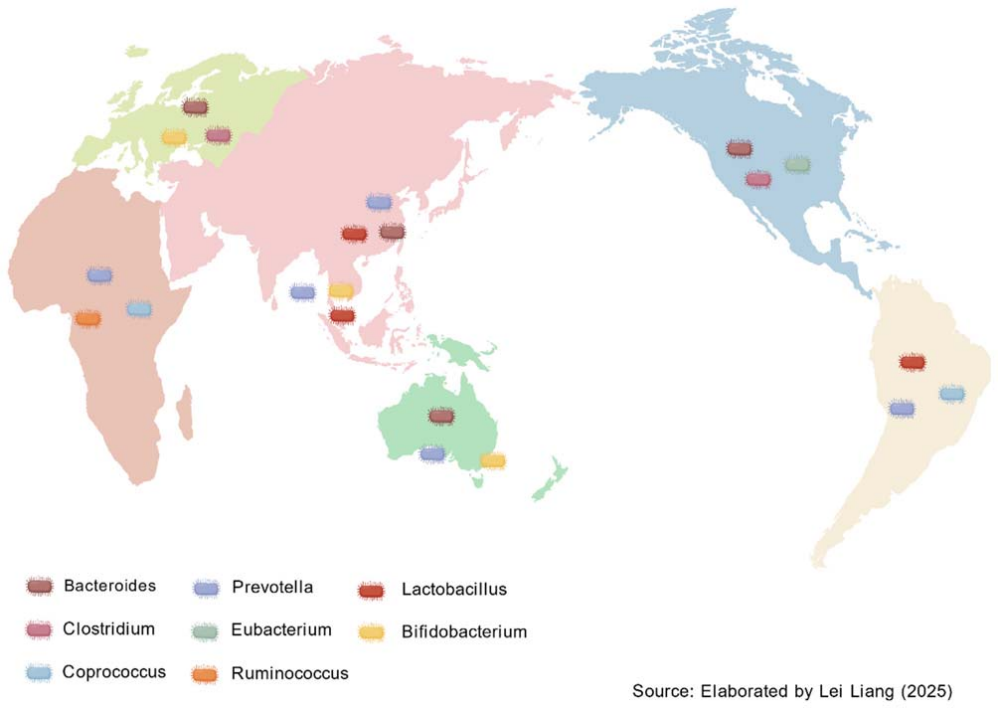
The top three genus-level bacterial communities in the intestines of major ethnic groups across different regions of the world.

The “Ethnicity-Gut Microbiota” phenomenon refers to the significant characteristic differences in the composition, structure, and function of gut microbiota observed among distinct ethnic groups. These differences arise from variations in genetic background, living environments, dietary habits, cultural traditions, and other related factors. This concept emphasizes the association between ethnicity—viewed as a composite variable encompassing genetics, environment, behavior, and other dimensions—and gut microbiota. The formation of human populations has been shaped by long-term processes of migration, isolation, and environmental adaptation, while gut microbiota variations are driven by the combined effects of multiple factors (Figure 1).

## 2 Factors influencing the “Ethnicity-Gut Microbiota” relationship

Extensive research on ethnicity and gut microbiota has demonstrated that the connection between the two is not solely determined by genetics; numerous other factors also play crucial roles in shaping this relationship. Chen et al. (2016) analyzed the gut microbiomes of 139 Americans, 368 Chinese individuals, and 760 Europeans, identifying genetics, diet, and living environments as the primary factors influencing gut microbiome diversity across different ethnic groups. Through a comprehensive review of relevant literature, we have concluded that three key factors—genetic makeup, geographic environment, and lifestyle—collectively drive the differences in gut microbiome composition among various ethnic groups (Dwiyanto et al., 2021a,b).

### 2.1 Genes

Over the course of long-term evolution, distinct ethnic groups have developed unique genetic variations (Friligkou et al., 2024). Ancestral genetic factors significantly influence human genome-wide gene expression and DNA methylation (Natri et al., 2020). For instance, studies have shown that Japanese Americans exhibit higher levels of DNA methylation—primarily in promoter regions—compared to European Americans (Song et al., 2021). A joint study by the U.S. Gut Project and the Human Microbiome Project, which analyzed the gut microbiomes of 1,673 participants, found that while factors such as gender, age, and body mass index (BMI) notably impact gut microbiota, racial genetics play a decisive role (Brooks et al., 2018).

**Figure 2:**
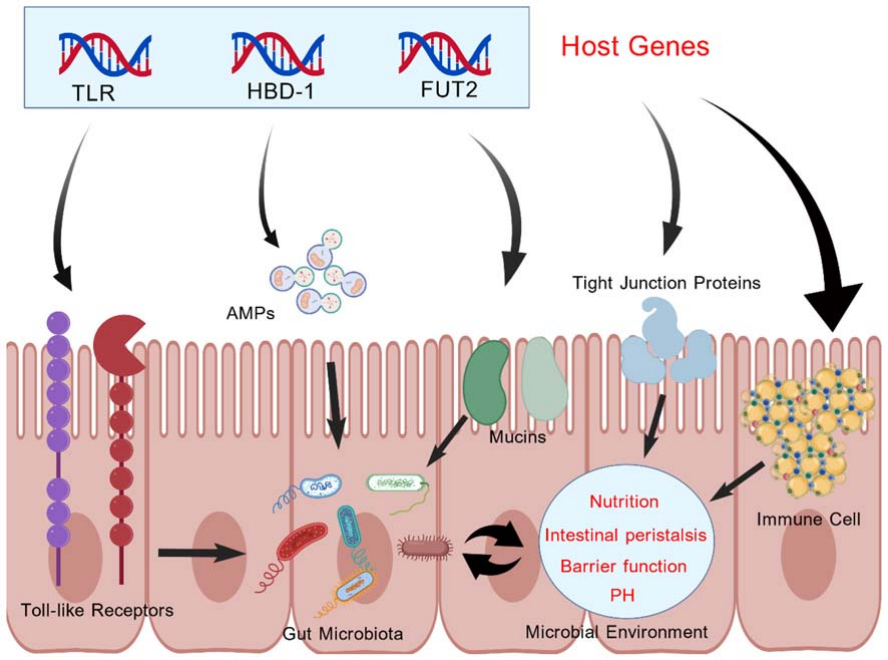
Host genes (TLR, HBD-1, FUT2) drive the expression of Toll-like receptors (microbial pattern recognition), antimicrobial peptides (pathogen suppression), and mucosal fucosylation (symbiotic bacterial nutrition supply), thereby influencing the composition and structure of the human gut microbiota. Additionally, host genes can influence tight junction proteins and immune cells in the gut, thereby affecting the composition of the gut microbiota by altering the microenvironment of the gut microbiota.

Host genetics can influence the composition of specific gut microbial communities (Liang et al., 2025). This is evident in identical twins, who show a higher degree of similarity in gut microbiota compared to non-identical individuals (Stewart et al., 2005; Lim et al., 2014). Host genes primarily affect gut microbiota through two major pathways: direct regulation of host molecular expression and indirect shaping of the intestinal microenvironment (Figure 2).

#### 2.1.1 Direct effect pathway: host-encoded molecules directly act upon the microbiota

Host genes directly screen and regulate the composition and activity of gut microbiota by encoding immune molecules (e.g., Toll-like receptors), mucins, and antimicrobial peptides (AMPs; Parizadeh and Arrieta, 2023; Pepke et al., 2024).

##### 2.1.1.1 Immune-related molecules

Genes of the Toll-like receptor (TLR) family are critical molecules linking host genetics, gut microbiota, and immune health, and represent classic targets for host genetics-microbiota interactions (Chen et al., 2024). As key genes encoding pattern recognition receptors (PRRs), their products specifically recognize cell wall components and nucleic acid fragments of gut microbiota, triggering immune responses to regulate microbial balance and maintain intestinal health (Glavan et al., 2016; Carvalho et al., 2012).

Different TLR family members recognize distinct pathogen-associated molecular patterns (PAMPs) from bacteria. For instance, a loss-of-function mutation in the TLR5 gene (*rs5744168*) impairs the host's ability to recognize bacterial flagella, leading to increased abundance of pro-inflammatory bacteria (e.g., *Escherichia coli*) in the gut (Oh et al., 2014; Chassaing and Gewirtz, 2018; Vijay-Kumar et al., 2010). This mutation occurs in approximately 10% of European populations but less than 1% of sub-Saharan African populations (Auton et al., 2015). Global population metagenomic studies further confirm that microbial traits associated with this mutation—such as elevated *Escherichia coli* levels—are more prominent in European populations, validating the impact of host genes on gut microbiota (Yatsunenko et al., 2012). Similarly, mutations in the TLR4 gene reduce the ability to recognize lipopolysaccharides from Gram-negative bacteria. These mutations are significantly more frequent in East Asian populations than in Europeans, which may contribute to differences in the abundance of certain Gram-negative bacteria (e.g., *Bacteroides*) between these groups (Ciesielska et al., 2021; Silva et al., 2022; Arbour et al., 2000).

##### 2.1.1.2 AMPs

Intestinal epithelial cells and immune cells express gene-encoded AMPs, such as defensins and lysozyme (Neurath et al., 2025). AMPs recognize differences in bacterial cell membranes, preferentially disrupting the membranes of Gram-negative bacteria (e.g., *Escherichia coli*) or Gram-positive bacteria (e.g., *Staphylococcus aureus*), leading to their lysis and death (Rizzetto et al., 2023). Notably, AMPs cause less damage to beneficial gut bacteria like *Lactobacillus* and *Bifidobacterium*, thereby impacting microbial community structure (Zong et al., 2020).

Common regulatory genes for AMPs include β-defensin 1 (HBD-1) and cathelicidin (LL-37; Srivastava et al., 2021). HBD-1 is widely expressed in mucosal tissues such as the intestines and respiratory tract (Kelly et al., 2013). Research shows that baseline intestinal HBD-1 expression levels are generally higher in Asian populations than in some European populations (Jurevic et al., 2002). This also helps explain why *Lactobacillus* and *Bifidobacterium* are typically more abundant in the gut of Asian populations (Wang et al., 2015).

##### 2.1.1.3 Mucins

Mucins secreted by intestinal epithelial cells are synthesized under the regulation of specific genes, forming the intestinal mucus layer (Neurath et al., 2025). The mucus layer not only acts as a physical barrier but also has surface glycans that serve as nutrients for gut microbiota (Luis and Hansson, 2023). Different mucin structures selectively nourish distinct microbial communities, influencing their colonization (Luis and Hansson, 2023).

The O-glycan structure of mucins exhibits high racial specificity (Probert et al., 1995). Its synthesis is regulated by glycosyltransferase genes, and the polymorphism of these genes varies significantly across populations (Lopera-Maya et al., 2022; Hansson, 2020). For example, fucosylated glycans, secreted via the fucosyltransferase 2 (FUT2) gene, are key nutrients for gut microbiota (Kamioka et al., 2022). The FUT2 gene deletion mutation (e.g., *rs601338*) occurs significantly more frequently in East Asian populations than in European populations (Pang et al., 2001). This enzyme also catalyzes the attachment of fucose molecules to glycoproteins or glycolipids on the cell surface, participating in the formation of important glycan structures such as the H antigen in the ABO blood group system, thereby influencing blood group expression (Kim J. et al., 2023). According to the NCBI database entry “Antigens of the ABO Blood Group,” the frequency of blood type A in Caucasians (the main ethnic group in Europe) is 43%, while it is only 28% in Asians and 27% in Black people. Additionally, the proportion of Native Americans with blood type O is 54.6% (Garratty et al., 2004), and among indigenous peoples in Mexico and the Amazon region, the prevalence of blood type O generally exceeds 90% (Sánchez-Boiso et al., 2011; Barjas-Castro et al., 2003). The differential expression of the FUT2 gene affects the composition and structure of gut microbiota. Specifically, individuals with blood type A have a significantly higher abundance of *Akkermansia muciniphila* in their gut microbiota (Jensen et al., 2024), while individuals with blood type O face a higher risk of colonization by certain pro-inflammatory bacteria, such as *Helicobacter pylori* (Chakrani et al., 2018).

#### 2.1.2 Indirect effect pathway: host genes shape the gut microenvironment

Host genes indirectly alter the gut's physical, chemical, and nutritional environment by regulating the host's physiological functions, thereby influencing microbial survival and metabolism. For example, these differential genes can indirectly affect dietary preferences by modifying the host's tolerance to specific foods (e.g., lactose or alcohol) or taste preferences, ultimately providing microbiota with distinct substrates (e.g., lactose or dietary fiber; Rai et al., 2025).

Additionally, genes regulate the arrangement of intestinal epithelial cells and the expression of tight junction proteins (e.g., occludin and claudin), determining intestinal barrier integrity (Floor et al., 2025). The strength of intestinal barrier function influences microbial colonization sites and dictates whether microbiota or their products enter the bloodstream—triggering immune responses that, in turn, affect microbiota (Takiishi et al., 2017).

Finally, immune-related genes (e.g., HLA genes and cytokine genes) determine the developmental maturity and response patterns of the host's immune system (Navid et al., 2025). Significant differences in immune-related gene expression exist among distinct ethnic groups (De Silvestri et al., 2019; Kock et al., 2025). A mature immune system provides a stable environment for symbiotic microbiota by eliminating pathogenic microbes and maintaining immune tolerance, thereby indirectly shaping microbial community structure (Donald and Finlay, 2023).

In summary, genetic differences among ethnic groups can influence the abundance of specific microorganisms in the gut and exert effects on the host's metabolism (Goodrich et al., 2014). Interestingly, although the influence of host genes on gut microbiota is widely recognized, the extent to which they play a dominant role has long been a subject of debate. For example, a study conducted in Israel involving 1,046 individuals of diverse ethnicities found that host genetics had a minimal impact on gut microbiota composition, whereas geographical factors exerted a more significant influence on fecal microbiome composition (Rothschild et al., 2018).

### 2.2 Geography

**Figure 3:**
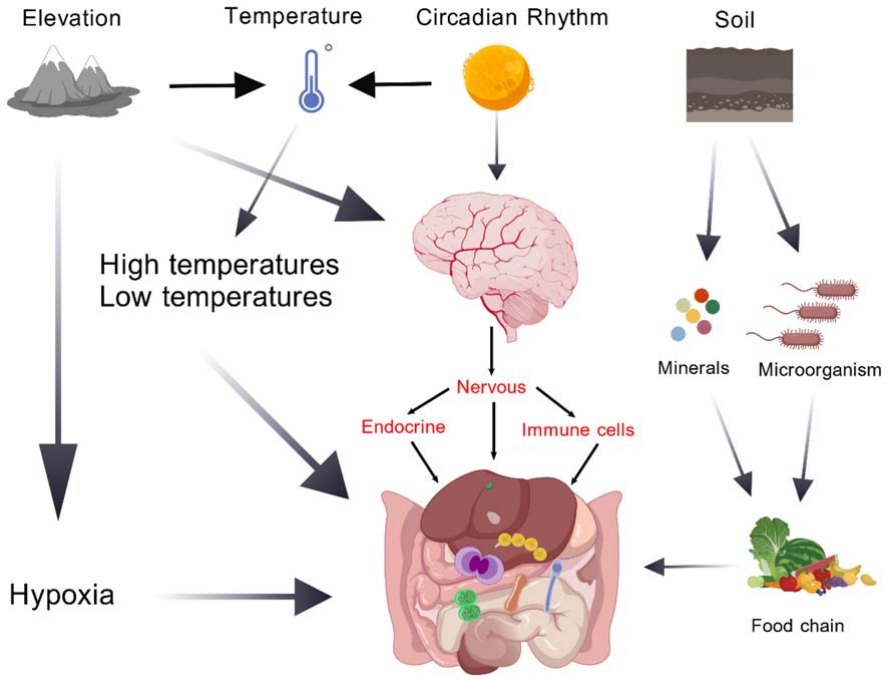
This diagram illustrates the multi-layered regulatory network linking environmental factors to host intestinal homeostasis. Elevation induces hypoxia, which directly alters intestinal mucosal perfusion and microbial metabolic activity. Temperature, modulated by both elevation and circadian rhythm, acts as a key environmental cue to shape gut microbial composition and host energy metabolism. Circadian rhythm further synchronizes intestinal function via neuro-endocrine-immune axes: neural signals regulate gut motility, endocrine hormones modulate barrier integrity, and immune cells fine-tune mucosal immune tolerance. Additionally, soil-derived minerals and environmental microorganisms enter the gastrointestinal tract via the food chain, directly influencing gut microbial colonization and community structure.

A variety of factors contribute to geographical variations, including elevation, soil composition, sunlight duration, and climate. A Chinese study involving 394 healthy individuals from seven ethnic groups (Bai, Hui, Korean, Miao, Mongol, Tibetan, and Uyghur) across seven cities found that geographical factors have a significant impact on the composition and diversity of gut microbiota (Lin et al., 2020). An Italian study, after controlling for factors such as gender, BMI, physical activity, diet, smoking, and drinking habits, also detected significant differences in gut microbiota among individuals of the same ethnicity living in different geographic locations (Fontana et al., 2019). Another study analyzed the gut microbiomes of 143 elementary school students from the Han, Hui, and Tibetan ethnic groups in Tibet, China. After controlling for geographical and environmental factors and ensuring no significant dietary differences, it was found that racial factors only influenced the α-diversity of gut microbiota, while no significant differences were observed in β-diversity (Liu et al., 2020). These findings collectively confirm that geographical factors contribute more significantly to differences in gut microbiota among various ethnic groups (Figure 3).

#### 2.2.1 Elevation

At different altitudes, temperature, atmospheric pressure, and air oxygen content vary significantly. Low-oxygen conditions can trigger intestinal inflammation, increase intestinal permeability, and alter the structure of gut microbiota (Wang J. et al., 2023; Han et al., 2022). The hypoxic state caused by low atmospheric pressure at high altitudes can significantly reduce the relative abundance and diversity of gut microbiota (Du et al., 2022). For example, an analysis of fecal samples from seven mountaineers participating in a 47-day expedition in the Nepalese Himalayas revealed a significant decrease in the abundance of *Bifidobacterium, Atopobium, Eggerthella*, and *Corynebacterium*, while the abundance of opportunistic pathogens such as *Proteobacteria* and *Enterobacteriaceae* increased markedly (Kleessen et al., 2005). Another study found that as altitude rises, the number of anaerobic bacteria and facultative anaerobes in the body increases significantly, while the number of aerobic bacteria decreases significantly (Adak et al., 2013). Interestingly, in this study, strictly anaerobic bacteria such as *Bifidobacterium, Bacteroides*, and *Lactobacillus*, as well as facultative anaerobes like *Clostridium perfringens* and *Peptostreptococcus*, showed a positive growth trend with increasing altitude. Regardless of the specific patterns, all research findings highlight the impact of altitude on the human gut microbiota.

Hypoxia reduces oxygen delivery to the intestinal mucosa, rendering the intestinal lumen in a more pronounced hypoxic state and inducing alterations in the intestinal microenvironment (Wu et al., 2021). Different bacterial genera within the gut microbiota display vastly different tolerances to oxygen. For example, the phylum Firmicutes exhibits greater tolerance to hypoxic environments than the phylum Bacteroidetes. Such differences can drive shifts in the dominant phyla of the microbiota, ultimately altering its overall composition (Lee et al., 2022; Cai et al., 2023).

Additionally, low temperatures at high altitudes trigger increased energy demands in the human body, requiring the gut microbiota to participate more efficiently in food digestion and energy absorption (Chevalier et al., 2015). To meet this demand, microbial populations that produce short-chain fatty acids (e.g., butyric acid and propionic acid)—including genera such as *Faecalibacterium* and *Eubacterium*—undergo an increase (Chevalier et al., 2015; Wang et al., 2024). This is because short-chain fatty acids directly energize intestinal cells, helping the body cope with energy expenditure during cold exposure (Xiao and Kang, 2020).

Finally, high altitude acts as a stressor for the human body, triggering a stress response that impacts intestinal function via the neuro-endocrine-immune network, thereby modifying the microbiota (Chen et al., 2012). Under stress, the body secretes hormones like cortisol, which slow intestinal motility and prolong the residence time of food in the intestines (Czimmer and Tache, 2017). Changes in food residence time affect microbial nutrient acquisition, while stress also increases intestinal permeability, enabling microbial metabolites to enter the bloodstream more readily. This, in turn, further regulates microbial balance (Minnebo et al., 2023; Collins et al., 2012).

#### 2.2.2 Soil

Differences in soil microbial structure and inorganic mineral content can lead to variations in gut microbiota (Peters et al., 2021; Zhou et al., 2022). The primary pathways through which soil exerts this influence are via water sources and plant-based foods. Growing up in different soil types (e.g., desert, grassland, forest) has a significant impact on the composition of gut microbiota in adulthood, and some characteristic microorganisms can persist for a long time even after environmental changes (Liu et al., 2021). Soil microorganisms can enter the human gut through the food chain (e.g., inadequately washed vegetables) or direct contact, thereby affecting the structure of the gut microbial community (Richard and Sokol, 2019). Beneficial bacteria such as *Bifidobacterium* and potential pathogens like *Salmonella* in the soil can directly participate in regulating the balance of the intestinal microbiota (Blum et al., 2019).

In addition, soil minerals can enter the food chain through plant absorption. Heavy metals such as arsenic and mercury, as well as persistent organic pollutants (POPs) in contaminated soil, can enter the human body through the food chain and disrupt the stability of gut microbiota (Wang H. T. et al., 2021; Andrade et al., 2023). Research indicates that microorganisms in arsenic-contaminated soil can alter the metabolic functions of gut microbiota, thereby increasing the host's susceptibility to diseases (Wang H. T. et al., 2021). On the other hand, plant-based foods rich in selenium can alleviate pathological damage to the gut, promote the colonization of *Bifidobacterium* and *Lactobacillus* in the mouse intestine, and inhibit the growth of *Escherichia* and *Enterococcus* in the gut (Yang et al., 2025). Furthermore, soil minerals such as calcium and magnesium can promote the growth and metabolism of beneficial gut bacteria (Wang et al., 2022; Piuri et al., 2021).

#### 2.2.3 Circadian rhythm

The Earth's rotation creates a day-night cycle, and the duration of day and night varies across different latitudes. The equatorial region receives the most sunlight, while high-latitude areas near the poles experience polar days that can last up to six months. The suprachiasmatic nucleus (SCN) at the base of the human hypothalamus perceives external light information (light/dark cycles) through the retina and regulates the body's internal environment (Hastings et al., 2020). Research has found that the relative abundance of Firmicutes and Actinobacteria increases during the day, while that of Bacteroidetes increases at night (Mortaş et al., 2020). Approximately 10% of the human gut microbiota exhibits circadian rhythmicity (Thaiss et al., 2014), with microorganisms such as *Parabacteroides, Bulleida, Enterobacteriaceae, Roseburia, Ruminococcus*, and *Veillonella* showing more prominent rhythmic patterns (Kaczmarek et al., 2017).

Narrowband ultraviolet B (NB-UVB) in sunlight can alter the composition of gut microbiota and increase its diversity (Bosman et al., 2019). In this study, 12 healthy women underwent three full-body NB-UVB treatments over 1 week. The results showed that exposure to NB-UVB significantly increased both alpha and beta diversity of the subjects' gut microbiomes. At the family level, the abundance of microorganisms such as Lachnospiraceae, Rikenellaceae, Ruminococcus, and Desulfobacteraceae increased to varying degrees (Bosman et al., 2019). Sunlight exposure also promotes the synthesis of vitamin D in the skin. Vitamin D is not only crucial for bone health but also regulates intestinal immune function, strengthens the gut barrier, inhibits the growth of harmful bacteria, and promotes the proliferation of beneficial bacteria (Kamweru and Tindibale, 2016; Franco and McCoy, 2024). Insufficient sunlight exposure impairs vitamin D synthesis, weakens intestinal immunity, and increases the risk of intestinal infections (Pham et al., 2021).

The influence of circadian rhythms on gut microbiota is not direct but is mediated through the brain-gut axis (Soliz-Rueda et al., 2025). Specifically, circadian rhythms modify the survival environment of gut microbiota through three major pathways—neuroendocrine, endocrine, and immune—by altering factors such as intestinal motility, pH levels, nutrient supply, and barrier function, thereby shaping the structure and function of the microbiota (Bonaz et al., 2018; Powell et al., 2025; Brooks et al., 2021).

The neural pathways of the brain-gut axis are centered on the vagus nerve and sympathetic nerve, whose activity exhibits significant circadian fluctuations. During the day, the activity of the vagus nerve increases through the release of neurotransmitters such as acetylcholine (Chrobok et al., 2022), which accelerates intestinal peristalsis, increases intestinal mucus secretion and digestive enzyme secretion, and thus alters the ratio of nutrients in the gut (Specian and Neutra, 1980; Gautam et al., 2005). This indirectly selects microbial communities that prefer specific nutrients and reduces the colonization of harmful bacteria on the intestinal mucosa. At night, the activity of the sympathetic nerve relatively increases, releasing neurotransmitters such as norepinephrine to inhibit intestinal peristalsis and regulate the tight junctions of intestinal epithelial cells (Hanman et al., 2019).

Under the regulation of the SCN, cortisol secreted by the adrenal glands also exhibits the classic “morning high, evening low” pattern (O'Byrne et al., 2021). Cortisol promotes the secretion of bicarbonate by intestinal epithelial cells (Sellin and Field, 1981). During the daytime, when cortisol levels are elevated, the intestinal pH becomes slightly higher. This physiological shift is accompanied by two notable changes in the gut microbiota: a significant reduction in the abundance of *Bifidobacterium* and a concurrent enrichment of *Ruminococcus gnavus*—a bacterium known to degrade cortisol (Zhang M. et al., 2024). Nighttime pH levels decrease, inhibiting the proliferation of certain harmful bacteria (such as *Helicobacter pylori*), further demonstrating the role of circadian rhythms in regulating gut microbiota through hormonal changes (Rigaud et al., 1988; Sjöström and Larsson, 1996).

Moreover, as the body's largest immune organ, the gut houses 70% of immune cells (Wiertsema et al., 2021). Circadian rhythms can directly regulate immune cells in the intestinal lamina propria via the sympathetic nervous system (Teng et al., 2019). During the day, immune cell activity increases, enabling the clearance of pathogenic bacteria entering the intestines with food and preventing them from competing with the native microbiota (Thaiss et al., 2014; Schiller et al., 2021); at night, immune cell activity decreases, reducing immune attacks on the gut microbiota and maintaining microbial homeostasis (Teng et al., 2019; Godinho-Silva et al., 2019). Additionally, anti-inflammatory cytokines such as IL-10 are secreted in greater quantities at night, protecting beneficial bacteria from immune-mediated damage (Tuganbaev et al., 2020); pro-inflammatory cytokines (such as TNF-α) exhibit slightly higher secretion during the day, primarily targeting foreign pathogens (Rahman et al., 2015). Disruption of circadian rhythms can lead to an imbalance in the diurnal secretion of pro-inflammatory factors, resulting in a decline in microbial diversity (Lin Y. et al., 2024). In summary, different circadian rhythms, driven by geographical differences in sunlight duration, influence the composition of gut microbiota through the brain-gut axis, adding another layer of complexity to the “Ethnicity-Gut Microbiota” relationship.

#### 2.2.4 Temperature

Environmental temperatures also vary at different elevation levels and periods of sunlight exposure, creating distinct thermal conditions that can affect gut microbiota. High temperatures increase the host's metabolic rate and suppress appetite, reducing food intake and thereby altering the structure of the gut microbiota (Wu et al., 2024). Low-temperature exposure leads to significant changes in gut microbiota β-diversity, with a marked increase in the abundance of *Paraclostridium*, which is associated with the host's thermogenesis capacity (Khakisahneh et al., 2020). Seasonal temperature fluctuations also influence microbial community structure. During winter's low-temperature environment, gut microbiota α diversity and Firmicutes abundance reach their peak, potentially reflecting the host's energy storage mechanisms to withstand cold conditions; during summer heatwaves, the abundance of *Verrucomicrobia* and *Spirochaetes* increases, suggesting a shift in microbial community function toward carbohydrate metabolism and energy utilization (Williams et al., 2023; Matsumoto et al., 2023). Overall, both high and low temperatures can negatively impact the human gut microbiota. Different gut bacteria have distinct temperature ranges for survival, and environmental temperatures can directly alter the structure of the gut microbiota, further contributing to ethnic differences in gut microbial profiles when combined with geographical variations in temperature.

### 2.3 Lifestyle

**Figure 4:**
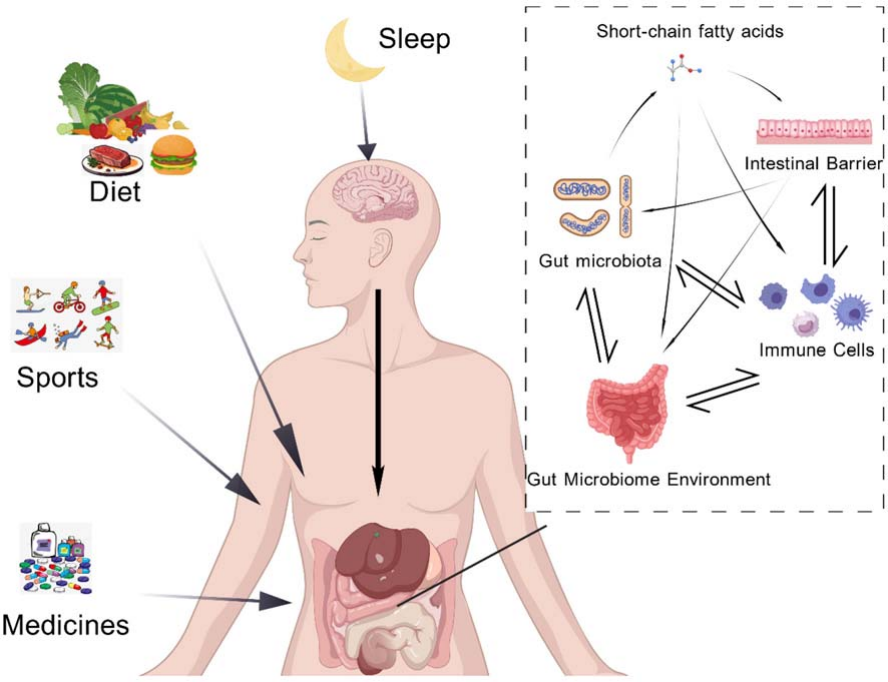
Extrinsic factors including dietary composition, physical activity, sleep quality, and pharmacotherapy directly and indirectly shape the taxonomic composition and metabolic activity of the gut microbiota. Dietary fiber undergoes microbial fermentation to produce short-chain fatty acids (SCFAs), which function as key signaling molecules performing three roles: (1) Enhancing intestinal barrier integrity by promoting tight junction protein expression; (2) Regulating immune cell polarization and cytokine secretion to maintain mucosal immune tolerance; (3) Engaging in bidirectional interactions with the gut microbiota and intestinal epithelial cells to stabilize the intestinal microbial environment.

A longitudinal cohort study examining gut microbiota diversity in 106 Chinese, Malaysian, and Indian infants from the same geographic location identified breastfeeding patterns and ethnicity as key factors influencing the development of gut microbial community composition (Xu et al., 2020). The effects of breastfeeding patterns persist until 6 and 3 months, respectively, primarily influencing the diversity and temporal colonization patterns of the genera *Bacteroides* and *Bifidobacterium*. The influence of ethnicity becomes apparent after 3 months of age, suggesting that lifestyle factors associated with ethnicity begin to play a role in shaping gut microbiota at an early stage (Xu et al., 2020). Another study demonstrates that among individuals of the same ethnic group residing in the same geographic location, variations in lifestyle—specifically the contrast between nomadic and sedentary modes—can lead to the development of distinctly different gut microbiomes (Keohane et al., 2020). Different ethnic groups exhibit distinct lifestyles due to cultural differences, which may be one of the primary reasons for the differences in gut microbiota among different ethnic groups (Sanchez-Johnsen et al., 2019; Figure 4).

#### 2.3.1 Diet

Diet, as one of the primary factors contributing to differences in lifestyle among various ethnic groups (Gray et al., 2018), exerts a significant influence on the composition and structure of the gut microbiota (Ross et al., 2024). A study of Hispanic and Latino populations in the United States revealed that after immigrating to the country, they experienced reduced biodiversity in their gut microbiome, diminished fiber-degrading capacity of their gut microbiota, and significant alterations in the composition and structure of their individual gut microbiomes (Wang Z. et al., 2021). Further analysis reveals that this phenomenon is associated with Westernized dietary habits during post-migration cultural adaptation, highlighting the profound impact of diet on gut microbiota (Wang Z. et al., 2021). A large prospective study by Borrello et al. (2022) revealed that among five racial groups—African Americans, Japanese Americans, Latinos, Native Hawaiians, and Caucasians—the abundance of 12 genera of gut microbiota was influenced by one or more of the eight dietary factors examined in the study. A consistent dietary pattern can also regulate and offset differences in gut microbiota composition across racial groups, suggesting that diet may be a modifiable factor in shaping gut microbiota (Borrello et al., 2022).

The Mediterranean diet refers to the eating habits of southern European countries bordering the Mediterranean Sea, such as Greece, Spain, France, and southern Italy. The diet primarily consists of vegetables, fish, whole grains, legumes, and olive oil. It regulates the structure of the gut microbiota through dietary fiber, extra virgin olive oil, and unsaturated fatty acids. The Mediterranean diet increases the abundance of probiotics such as *Bacteroides, Bifidobacteria, Eubacterium, Faecalibacterium prausnitzii*, and *Clostridium leptum*, while reducing the abundance of bacterial groups including Firmicutes and *Blautia* species (Barber et al., 2023). These probiotics enhance the production of short-chain fatty acids such as butyrate, regulating epithelial barrier function and intestinal mucosal epithelial cells through signaling pathways like G protein-coupled receptors or histone deacetylases, thereby influencing systemic immunity (Mann et al., 2024). Additionally, butyric acid can directly influence the differentiation of phagocytes, B cells, plasma cells, and regulatory T cells to counteract inflammatory responses (Mann et al., 2024). A growing body of research confirms that the Mediterranean diet can influence gut microbiota to reduce the risk of malignant tumors and cardiovascular disease, improve cognitive function, lower metabolic risks associated with diabetes, obesity, and metabolic syndrome, and extend lifespan, highlighting the potential health benefits of this diet through its effects on gut microbiota (Muscogiuri et al., 2022; Salim et al., 2023; Puljiz et al., 2023; Dimba et al., 2024; Andreo-López et al., 2023).

Western diets, exemplified by the traditional American diet, are characterized by high energy density, high fat content, low fiber content, and high salt content. The lack of fiber and prebiotics essential for promoting the development and diversity of a healthy gut microbiota has been linked to a range of chronic diseases and inflammatory processes, including obesity, type 2 diabetes, cardiovascular disease, and certain cancers (Clemente-Suárez et al., 2023). Western diets disrupt the ecological diversity of the gut microbiome, leading to reduced abundance of probiotics and overgrowth of harmful bacteria (Jawhara, 2023). Swiatecka et al. (2011) indicate that Western diets increase the abundance of bacteria such as *Bacteroides, Alistipes*, and *Bilophila*, while decreasing the abundance of butyrate-producing bacteria, including *Ruminococcus, Lactobacillus, Roseburia, Bifidobacterium*, and *Eubacterium*. Dysbiosis of the gut microbiota compromises intestinal barrier function, increases intestinal permeability, and promotes bacterial translocation, thereby triggering systemic inflammation (Majumder and Bano, 2024). Additionally, dysbiosis of the gut microbiota can regulate the differentiation and activation of immune cells, modulate immunoglobulin secretion, and influence cytokine production, thereby affecting immune system function, further demonstrating the negative impact of Western diets on gut microbiota and overall health (Prame Kumar et al., 2023).

Plant-based diets, primarily encompassing vegetarian and lacto-ovo vegetarian diets, are gaining increasing popularity worldwide due to their health benefits (Craig et al., 2021). Dietary polyphenols are plant-based natural chemicals found in tea, coffee, grains, vegetables, and fruits (Shah et al., 2024). This is a complex and diverse class of organic molecules characterized by hydroxylated phenyl groups, typically categorized into flavonoids and non-flavonoids, and found in very low quantities in Western diets (Pérez-Jiménez, 2024). Dietary polyphenols exhibit antioxidant, anti-diabetic, anti-cancer, neuroprotective, anti-inflammatory, cardioprotective, antibacterial, and anti-adipogenic properties (Tresserra-Rimbau, 2020). Additionally, dietary polyphenols, acting as human prebiotics, regulate the ecological structure of the gut microbiota (Luca et al., 2020; Davinelli and Scapagnini, 2022). Plant-based diets can increase the abundance of butyrate-producing probiotics such as *Lactobacillus, Prevotella*, and *Bifidobacterium*, thereby reducing the risk of chronic diseases, making them a promising dietary pattern for promoting gut health and overall wellbeing (Lares-Michel et al., 2023; Azad et al., 2018).

#### 2.3.2 Sports

Over thousands of years of human civilization's evolution, distinct lifestyles—hunting, nomadic herding, and agriculture—have shaped significant variations in athletic capabilities among different ethnic groups (Shephard and Aoyagi, 2009). The athletic prowess of African Black people, primarily hunters, and European whites, predominantly nomads, is generally considered superior to that of Asian yellow-skinned peoples, who are mainly agriculturalists (Outram et al., 2018). Throughout their long history of hunting and nomadic life, black and white populations engaged in more frequent physical activity compared to yellow-skinned populations, who primarily practiced agriculture. Prolonged aerobic exercise enhances human endurance and metabolic capacity (Uchida et al., 2023). Although differences in lifestyles are gradually diminishing in today's globalized world, millennia of distinct living patterns have shaped our genetic makeup (Zilberman-Schapira et al., 2012). Basal metabolic genes, regulatory genes, and myogenic genes associated with skeletal muscle change with prolonged exercise habits, potentially influencing the interaction between exercise and gut microbiota (Mallett, 2025).

Existing research has established that athletic performance is influenced by gene expression (García-Giménez et al., 2024). Genes such as Alpha-Actinin 3, Angiotensin I-Converting Enzyme, Nitric Oxide Synthase 3, and Peroxisome Proliferator Activated Receptor Alpha (PPARA) determine human athletic ability (Zilberman-Schapira et al., 2012). Another systematic review also indicates that the expression of genes such as Adenosine Monophosphate Deaminase-1, Peroxisome Proliferator Activated Receptor gamma (PPARG), Peroxisome Proliferator-Activated Receptor gamma Coactivator 1 Alpha (PPARGC1A), and PPARA in skeletal muscle also determines an athlete's athletic aptitude (Balberova et al., 2021). A study in the United States found significant differences in PPARG gene expression between Caucasians and African Americans (Underwood et al., 2010). The Pro12Ala polymorphism in the PPARG gene is associated with racial factors (West et al., 2018). The expression level of PPARA is lower in African Americans than in Caucasians, suggesting that genetic differences among ethnic groups may contribute to variations in athletic performance and potentially influence the effects of exercise on gut microbiota (Shin et al., 2008).

Exercise can regulate and balance energy metabolism within the human body, thereby altering the composition of the gut microbiota. The gut microbiota observed in professional athletes differs significantly from that of sedentary individuals. Athletes exhibit more intense intestinal metabolic activity, with higher abundances of butyrate-producing probiotics such as *Anaerostipes hadrus, Clostridium bolteae, Faecalibacterium prausnitzii*, and *Roseburia hominis* within their microbiota (Sabater et al., 2023). A meta-analysis indicates that exercise significantly reduces the abundance of Bacteroidetes and increases the abundance of Firmicutes (Min et al., 2024). Exercise can prevent the onset of various diseases, such as major depression, Alzheimer's disease, obesity, cardiovascular disease, and gastrointestinal disorders, by altering the structure of the gut microbiota, highlighting the potential role of exercise as a modifiable lifestyle factor in promoting gut health and preventing disease (Wegierska et al., 2022; Souza et al., 2023; Cutuli et al., 2023; Jin et al., 2024; Hawley et al., 2025; Cancino Ramírez et al., 2025).

#### 2.3.3 Sleep

Sleep duration is closely linked to economic outcomes and exhibits a significant negative correlation (Stevens, 2015; Bapat et al., 2016). The more economically developed a region is, the more vibrant its nightlife tends to be, and the less sleep its residents generally get. Research indicates that non-Hispanic Asians, non-Mexican Hispanics/Latinos, and non-Hispanic Black people have shorter sleep durations than non-Hispanic Whites (Whinnery et al., 2014). Race and economic status jointly influence sleep duration (Ji et al., 2023). Interestingly, even within the same region, sleep duration varies across racial groups. A U.S. study of 4,522 participants found that African Americans slept less than whites, and this was associated with cardiovascular disease (Butler et al., 2020). Another large-scale, nationally representative cross-sectional survey in the United States involving 274,836 participants demonstrated significant differences in sleep duration among five racial groups—White, Black, AIAN (American Indian/Alaska Native), Asian, and Hispanic—and these differences were associated with all-cause mortality (Denney et al., 2023), emphasizing the importance of sleep duration as a factor contributing to health disparities among ethnic groups.

Short sleep duration reduces gut microbiota diversity and decreases the abundance of beneficial gut bacteria (Karl et al., 2023). In an animal study, acute sleep deprivation led to a significant decline in both α-diversity and β-diversity of mouse gut microbiota, with markedly increased abundance of *Enterobacter* and *Candidatus Arthromitus*, while *Muribaculum, Lactobacillus, Parasutterella*, and *Monoglobus* showed significantly reduced abundance (Yang et al., 2023). Clinical studies have found that individuals with insufficient sleep exhibit significantly higher abundance of *Pseudomonas* compared to those with normal sleep patterns, while the abundance of *Sutterella* is markedly lower than in the normal sleep group (Agrawal et al., 2021). Another study found that individuals with sleep deprivation for just two days exhibited an increased Firmicutes: Bacteroidetes ratio in their gut compared to those with normal sleep, with significantly elevated abundances of *Coriobacteriaceae* and *Erysipelotrichaceae*—both associated with obesity—and reduced *Tenericutes* abundance (Benedict et al., 2016). Short sleep duration disrupts the circadian rhythms of the gut microbiota, triggering dysbiosis that in turn exacerbates insomnia. This ultimately contributes to the development of diseases such as diabetes, chronic inflammation, and cardiovascular and cerebrovascular disorders, highlighting the bidirectional relationship between sleep and gut microbiota and their combined impact on health (Lin Z. et al., 2024).

#### 2.3.4 Health and pharmaceuticals

Typically, different ethnic groups exhibit variations in hygiene conditions due to geographical and economic differences (Kowal et al., 2023). The “Hygiene Hypothesis” suggests that improved sanitation reduces early microbial exposure, leading to impaired immune development and reduced gut microbiota diversity (Pfefferle et al., 2021). Compared to rural or non-industrialized communities, urban populations with improved sanitation conditions often exhibit lower microbial diversity, characterized by reduced *Prevotella* and increased *Bacteroides* (Yatsunenko et al., 2012). Similarly, De Filippo et al. (2010) observed higher *Prevotella* abundance and lower levels of gut inflammation markers in African rural children compared to European children, attributing this to differences in hygiene conditions and dietary fiber intake, further supporting the role of hygiene in shaping gut microbiota.

Additionally, pharmaceutical factors contribute to variations in hygiene conditions. Generally, better hygiene conditions correlate with greater exposure to diverse medications. Antibiotics exert the most significant impact on human gut microbiota. Their widespread use in highly hygienic regions disrupts the body's original intestinal microbial balance. Antibiotics reduce gut microbial diversity while promoting the growth of drug-resistant strains, thereby increasing susceptibility to infections. Frequent antibiotic use may even lead to dysbiosis and pose health risks (Finlay et al., 2021). In contrast, limited antibiotic exposure in low-hygiene regions preserves more ancestral microbial lineages (Williams et al., 2023). Different antibiotics exert distinct effects on the gut microbiota. Broad-spectrum antibiotics such as β-lactams and fluoroquinolones significantly reduce Bacteroidetes and Firmicutes while promoting the proliferation of antibiotic-resistant bacteria like *Enterococcus* and *Escherichia* (Schwartz et al., 2020; Ferrer et al., 2014). Antibiotic drugs targeting anaerobic bacteria (such as metronidazole) deplete beneficial anaerobic bacteria like *Faecalibacterium* and *Roseburia*, while increasing the abundance of pathogenic bacteria such as *Clostridioides difficile* and *Klebsiella pneumoniae* (Schwartz et al., 2020). Additionally, beyond antibiotics, medications such as glucocorticoids, non-steroidal anti-inflammatory drugs (NSAIDs), immunosuppressants, antidiabetic drugs, proton pump inhibitors, chemotherapeutic agents, and psychiatric medications can also influence changes in the structure of the gut microbiota (Su et al., 2025; Liang et al., 2023a; Weersma et al., 2020; Bjarnason and Rainsford, 2021), highlighting the need to consider pharmaceutical use when studying ethnic differences in gut microbiota.

Simultaneously, drugs specific to different ethnicities and regions exert varying effects on the gut microbiota. The aforementioned Western medicines—including antibiotics, glucocorticoids, non-steroidal anti-inflammatory drugs (NSAIDs), and immunosuppressants—are primarily chemically synthesized compounds with well-defined targets, yet they may disrupt the balance of the gut microbiota (Wu et al., 2023; Džidić-Krivić et al., 2023). In contrast, Chinese herbal medicines—originating from China—are natural, complex botanical formulations containing diverse active components like polysaccharides, flavonoids, and alkaloids. They target multiple pathways and predominantly promote the proliferation of beneficial gut bacteria (Wang L. et al., 2023; Che et al., 2022). Similarly, ethnic-specific pharmacologies such as Indian Ayurveda, African Traditional Medicine, and Native American Medicine also contribute to variations in ethnic microbial profiles, further complicating the “Ethnicity-Gut Microbiota” relationship and emphasizing the importance of considering cultural and regional differences in pharmaceutical use.

### 2.4 Others

We discussed how hygiene conditions and medications affect the gut microbiota, noting that the root cause lies in economic disparities. Economics profoundly shapes the composition and function of gut microbiota through multiple factors, including dietary patterns, healthcare resource allocation, and living environments (Moreno-Altamirano et al., 2024; He et al., 2018). This influence is not unidirectional but intertwined with health and disease, giving rise to variations across different ethnic groups.

The impact of religion and culture on gut microbiota is fundamentally mediated through a complex “diet-behavior-environment” pathway. Religious dietary restrictions and rituals shape specific nutritional intake patterns (Syromyatnikov et al., 2022). For instance, some religions prohibit the consumption of certain meats, which can influence the types of nutrients available to gut microbiota and thus alter their composition (Suratno et al., 2025; Purdel et al., 2023). Furthermore, cultural differences across ethnic groups encompass traditional foods, hygiene practices, and lifestyles, which influence microbial composition through long-term adaptive selection. These cultural and religious factors, combined with economic and geographical influences, further contribute to the diversity of gut microbiota among different ethnic groups.

## 3 The interrelationship of multiple factors

**Figure 5:**
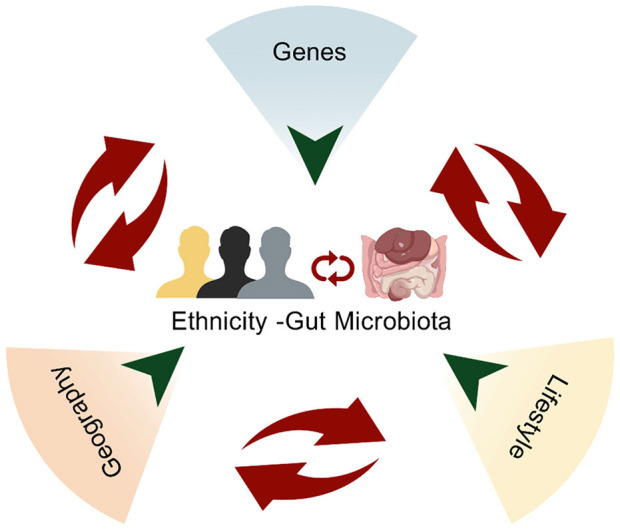
The three primary factors shaping the “Ethnicity-Gut Microbiota” relationship.

Although we have summarized above that differences in gut microbiota across ethnic groups may be driven by three major factors—genetics, geographic environment, and lifestyle—these factors do not influence gut microbiota independently. They interact with one another in complex ways, collectively shaping the composition of gut microbiota across ethnic groups. Understanding these interactions is crucial for unraveling the mechanisms underlying the “Ethnicity-Gut Microbiota” relationship (Figure 5).

### 3.1 Genes and geography

The interaction between geography and genes is one of the core driving forces behind biological evolution, population differentiation, and ecological adaptation. The geographic environment shapes an organism's gene pool through mechanisms such as natural selection and isolation, while genes determine an organism's ability to adapt to its geographic environment. Together, they form a dynamic “shaping-adaptation” relationship that jointly influences an organism's distribution, traits, and evolutionary pathways. For example, the low-oxygen, low-pressure environment of plateau regions selects for genes adapted to hypoxia (Huerta-Sánchez et al., 2014). The frequency of specific variants in the EPAS1 gene is significantly higher among Tibetan populations than in low-altitude groups (Hu et al., 2017). This gene reduces excessive red blood cell proliferation caused by hypoxia, preventing high-altitude illnesses such as pulmonary edema (Zhang et al., 2025). Meanwhile, Arctic-dwelling Inuit carry unique variants in the UCP1 gene that enable efficient fat-derived heat production, adapting them to extreme cold (Chouchani et al., 2019). These examples illustrate how geographic environments drive genetic adaptations, which in turn influence phenotypic traits.

Furthermore, circadian rhythms, shaped by geographic differences in sunlight duration, have influenced genes related to human skin pigmentation. In equatorial regions, intense ultraviolet radiation degrades folate, while specific variants in the MC1R gene promote melanin synthesis to protect folate, resulting in darker skin tones among African and South Asian populations (Aoki, 2002). In high-latitude regions with weaker UV radiation, variations in the SLC24A5 gene reduce melanin production, facilitating vitamin D synthesis in the skin (preventing rickets), resulting in fairer skin among Northern Europeans (Kim S. et al., 2023). These genetic adaptations to geographic differences in sunlight not only affect skin pigmentation but also have implications for vitamin D metabolism, which can further influence gut microbiota through immune regulation and other pathways (Soltys et al., 2020).

The interaction between geography and genetics not only shapes an organism's macroscopic traits but also profoundly influences the composition and function of the gut microbiota. As an “intermediate layer” mediating host-environment interactions, the diversity and structure of the gut microbiota are directly shaped by geographical factors—such as diet, climate, and microbial exposure—while also being indirectly regulated by host genetics through physiological mechanisms. These two elements form a dynamic synergistic relationship, jointly influencing the host's metabolism, immune function, and overall health status.

Research has identified CYB_1400 and Snas_0276 as genes unique to Europeans and Americans (Chen et al., 2016). Among these, CYB_1400 is a gene specific to *Synechococcus*, which can accumulate in the human body through the food chain, particularly in Atlantic and Mediterranean waters (Chaouni et al., 2023). Another gene, Snas_0276, encodes a unique sulfotransferase specifically expressed in *Stackebrandtia nassauensis*, which is associated with phosphate synthesis and carbohydrate metabolism. *Stackebrandtia nassauensis* is highly prevalent in the high-salinity Mediterranean Sea and accumulates in the gut via the food chain (Labeda and Kroppenstedt, 2005). The HemE gene, unique to Chinese and Europeans, encodes an important urapterin-1 decarboxylase in *Chlorobium phaeobacteroides*, primarily distributed in Asia and Europe (Repeta et al., 1989). Gura_R0049 is a transcription-related gene in *Geobacter uraniireducens*, renowned for its unique iron metabolism mechanism (Huang et al., 2018). *Geobacter uraniireducens* is predominantly found in deep-earth environments or heavy metal-contaminated areas. Historically, heavy metal smelting and production processes were concentrated in parts of Asia and Europe, with fewer heavy metal contamination sites in North America (Prakash et al., 2010). These findings suggest that geographic differences in the distribution of specific microorganisms and environmental factors can lead to the accumulation of unique microbial genes in the gut of different ethnic groups, further highlighting the interaction between genes and geography in shaping gut microbiota.

### 3.2 Genes and lifestyle

Genetic background and lifestyle factors (diet, exercise, sleep, etc.) do not independently influence human health and traits. Instead, they interact through complex mechanisms to jointly shape an individual's physiological state, disease risk, and phenotypic characteristics. Genes encoding proteins—such as enzymes, receptors, and transporters—they determine the body's metabolic capacity, tolerance thresholds, and physiological responses to lifestyle influences. For instance, variations in the LCT gene expression determine lactose digestion capacity, with individuals carrying certain variants able to digest lactose into adulthood, while others develop lactose intolerance (Oliveira et al., 2020). Genes like HTR1B, ADIPOR1, PPARGC1A, and CYP19A1 exhibit significant differences between Caucasian and African American cohorts. Among these, the ADIPOR1 gene is associated with smoking, the PPARGC1A gene with alcohol consumption, and the CYP19A1 gene with dietary intake and BMI. Furthermore, the ADIPOR1 gene is linked to multiple factors (Edwards et al., 2012), indicating the complexity of gene-lifestyle interactions.

Lifestyle influences gene expression through epigenetic regulation, which involves modifications to DNA or associated proteins that do not change the underlying genetic sequence but can alter gene activity. Diet can affect gene expression and epigenetics by altering nutrient availability, cofactor supply, hormone levels, environmental cues, cellular signaling pathways, transcription factors, chromatin structure, DNA methylation, histone modifications, microRNA regulation, feedback loops, genetic variation, and gene-environment interactions. For example, a diet rich in methyl donors can influence DNA methylation patterns, thereby affecting the expression of genes related to metabolism and immune function (La Cava, 2019). Regular aerobic exercise activates the expression of the PGC-1α gene (a key gene in mitochondrial biogenesis), enhances its activity through epigenetic modifications (such as histone acetylation), and improves mitochondrial function and endurance (Neto et al., 2023). Furthermore, short sleep duration can regulate human circadian rhythm genes, including occludin (OCLN), brain and muscle ARNT-like 1 (BMAL1), and cryptochrome circadian regulator 1 (CRY1), disrupting normal circadian rhythms and potentially influencing gut microbiota (Yang et al., 2023).

The relationship between genes, lifestyle, and gut microbiota is not unidirectional but forms a dynamic, interactive network among “host genes—lifestyle—gut microbiota.” Genes shape the host's physiological traits (such as immune function and metabolic capacity), providing a “foundation for survival” for the microbiota. Diet, exercise, sleep, and other factors reshape microbial structure by directly altering the microbial environment or regulating host gene expression. Conversely, the gut microbiota influences host gene activity and the “actual effects” of lifestyle choices through its metabolic products. This intricate interplay profoundly impacts the diversity, stability, and function of the gut microbiota.

Dietary polyphenols, polysaccharides, and peptides can modulate gut microbiota composition. Through gut microbiota-produced hormones and neurotransmitters, they activate or inhibit corresponding neurons, acting on the vagus nerve to regulate sleep rhythms (Dinan et al., 2015). Meanwhile, the fermentation efficiency of dietary fiber by microbiota is regulated by host genes. For instance, individuals with functional FUT2 genes exhibit increased abundance of SCFA-producing bacteria like *Bifidobacterium* when consuming high-fiber diets, as their intestinal mucus layer provides more fermentation substrates—a response significantly greater than in FUT2-mutant individuals (Stine, 2021; Lei et al., 2022). “Persistent expression mutations” in the LCT gene (e.g., *rs4988235*) enhance lactose digestion. This mutation occurs in over 80% of Europeans but is significantly less prevalent in most East Asian and African populations (Ingram et al., 2009; Ma et al., 2025). This results in a higher abundance of lactose-degrading gut bacteria (e.g., *Lactobacillus, Bifidobacterium*) in Europeans. Conversely, lactose-intolerant East Asians consume less dairy, leading to greater enrichment of bacteria that degrade plant polysaccharides (e.g., *Prevotella*; Smug et al., 2014; Masoumi et al., 2021; Kok and Hutkins, 2018). These examples illustrate how genes and lifestyle interact to shape gut microbiota, with genetic variations influencing the response to lifestyle factors such as diet.

### 3.3 Geography and lifestyle

Geography and lifestyle form a deeply intertwined dynamic relationship. The geographical environment provides the fundamental framework for human life, shaping the details of daily existence such as diet, housing, occupation, and culture. In turn, human lifestyles continuously reshape the landscape through activities like production, consumption, and the transformation of nature. The gut microbiota serves as a crucial link between the external environment and human health. Its diversity, dominant bacterial species, and metabolic functions are indirectly shaped by geographical environments through lifestyle patterns, while simultaneously influencing human adaptability to these environments. This chain of interactions between geography, lifestyle, and gut microbiota, along with the gut microbiota's reciprocal influence on human adaptation to geographical environments, forms a unique ecological association.

In tropical regions characterized by high temperatures and abundant rainfall, diets primarily consist of heat-clearing and summer-relieving staples like rice and fruits. The gut microbiota features a higher proportion of *Bacteroides* and *Bifidobacterium*, which excel at breaking down dietary fiber. These bacteria convert fiber into short-chain fatty acids (such as butyrate), aiding the body in anti-inflammatory processes and maintaining the intestinal barrier (John et al., 2020; Ruggles et al., 2018). In cold regions, the diet relies heavily on livestock, featuring high-fat foods (fish, beef, lamb) and high-protein foods (dairy products). The gut microbiota in these populations exhibits a higher proportion of Firmicutes, which efficiently break down fats and proteins to generate energy, enabling the body to withstand the cold (De Filippo et al., 2010; Huus and Ley, 2021). In temperate monsoon regions with distinct seasons, diets shift with the seasons, leading to greater gut microbiota diversity (Ross et al., 2024). During the rainy season (high temperature and humidity), the Hadza consume more fresh plant fiber, resulting in an enrichment of gut bacteria that degrade complex carbohydrates and a significant increase in Bacteroidetes abundance. Conversely, during the dry season (low temperature and dryness), the proportion of Firmicutes rises (Davenport et al., 2014). These examples demonstrate how geographical differences in climate and food availability shape dietary patterns, which in turn influence gut microbiota composition.

Additionally, coastal regions boast abundant fishery resources, with residents predominantly engaged in fishing and seafood processing, resulting in a diet heavily reliant on marine products (Bosanac et al., 2016; Petrenya et al., 2018). The abundance of Bacteroidetes (such as *Bacteroides multiformis* and *Bacteroides fragilis*) and Actinobacteria (such as *Propionibacterium*) in these populations is significantly higher than in inland communities. These bacteria secrete proteases (such as trypsin-like enzymes) that break down seafood proteins into small peptide molecules and amino acids, while simultaneously preventing excessive protein accumulation that could lead to intestinal ammonia buildup (reducing the risk of intestinal odor and inflammation; Zhang J. et al., 2024; Berding et al., 2018). The interaction between geography and lifestyle on gut microbiota is fundamentally the result of long-term co-evolution between humans and their environment, with each factor influencing the other in a continuous cycle.

## 4 The significance of research on the “Ethnicity-Gut Microbiota” relationship

Research on the association between ethnicity and gut microbiota has emerged as a hot topic in interdisciplinary fields such as microbiome science, medicine, and anthropology in recent years. Its significance extends beyond fundamental understanding of human microbial diversity to practical applications in disease prevention and control, as well as precision medicine. Investigating the relationship between ethnicity and gut microbiota helps distinguish the relative contributions of genetic factors, geographic factors, and lifestyle to gut microbiota composition. It clarifies how multifactorial interactions shape the unique microbial structures characteristic of different ethnic groups, providing a theoretical foundation for understanding gut microbiota diversity and its underlying mechanisms. This knowledge is essential for advancing our understanding of human biology and the role of the gut microbiota in health and disease.

Significant racial disparities exist in the incidence and clinical manifestations of many diseases (Fan et al., 2022; Zakeri et al., 2024; Hassan et al., 2023). For example, Asian populations exhibit higher susceptibility to diabetes compared to European and American populations (Kwan et al., 2023), while African Americans demonstrate unique pathological features in obesity-related metabolic disorders (Lofton et al., 2023). As a key mediator of host-environment interactions, the gut microbiota may represent a crucial target for elucidating these disparities. This holds significant implications for deciphering the mechanisms underlying racial differences in disease. Gut microbiota enriched in specific racial populations may produce short-chain fatty acids (SCFAs) through dietary fiber metabolism, thereby influencing host inflammatory responses or metabolic balance and potentially correlating with disease risk. Research has found that Asian populations exhibit a higher abundance of *Akkermansia muciniphila* in their gut microbiota, which may be associated with their lower risk of obesity (Ang et al., 2021). Conversely, the enrichment of pro-inflammatory bacteria such as *Escherichia coli* in the guts of African Americans may exacerbate insulin resistance and increase the risk of type 2 diabetes (Dugas et al., 2018). Clarifying these associations could help identify microbial biomarkers for diseases, offering new directions for etiological research and potentially leading to the development of targeted prevention and treatment strategies.

Additionally, gut microbiota can influence drug activity through metabolic transformations, and racially specific microbiota may lead to variations in drug responses (Ford, 2025; Eliasson, 2006). Research on the “Ethnicity-Gut Microbiota” relationship can optimize drug efficacy and safety. For instance, a systematic review found Asians face higher risks of anticoagulant-related adverse drug reactions, while Black patients exhibit elevated risks of diabetes medication-related adverse events, and Caucasians are most frequently identified as having increased risks of opioid-related adverse drug reactions (Baehr et al., 2015). Targeted modifications to the gut microbiota structure in racial populations can influence the efficacy of these drugs and enhance their safety, potentially reducing health disparities associated with drug responses (Chen et al., 2022; Lim et al., 2023; Eitan et al., 2021).

Second, research on the “Ethnicity-Gut Microbiota” Relationship can guide public health strategies and aid in developing more precise disease prevention programs. Targeted interventions (such as dietary adjustments and probiotic supplementation) can be designed based on the distinct microbiome characteristics of different racial groups. For example, if a particular ethnic group has a lower abundance of beneficial bacteria associated with carbohydrate metabolism, probiotic supplements containing these bacteria or dietary recommendations to increase the intake of prebiotics that promote their growth could be implemented. Furthermore, combining racial background with microbiome features can identify biomarkers for disease risk prediction, enabling the construction of more accurate disease risk prediction models. For example, integrating the microbiome characteristics of Asian populations (such as *Fusobacterium abundance*) with genetic markers enables earlier identification of individuals at high risk for colorectal cancer, thereby improving screening efficiency and reducing mortality rates (Yao et al., 2021).

Finally, “Ethnicity-Gut Microbiota” research can advance studies on human evolutionary history and migration patterns, refining the evolutionary perspective on human gut microbiomes. The human gut microbiota, often termed the “second genome,” has evolved in close association with human migration and environmental adaptation (Parizadeh and Arrieta, 2023). Microbiomes across different ethnic groups may retain microbial characteristics from their ancestral regions. For instance, certain bacterial genera unique to the guts of Southeast Asian populations correlate with their ancestors' adaptation to plant-based diets after migrating from Africa to Asia (Lee et al., 2017). Meanwhile, the microbiomes of Native Americans still harbor co-ancestral bacteria shared with Eurasian populations, supporting the “Bering Strait migration” hypothesis (Moodley et al., 2021). This research not only provides insights into human evolution but also helps us understand the co-evolutionary relationship between humans and their gut microbiota.

**Figure 6:**
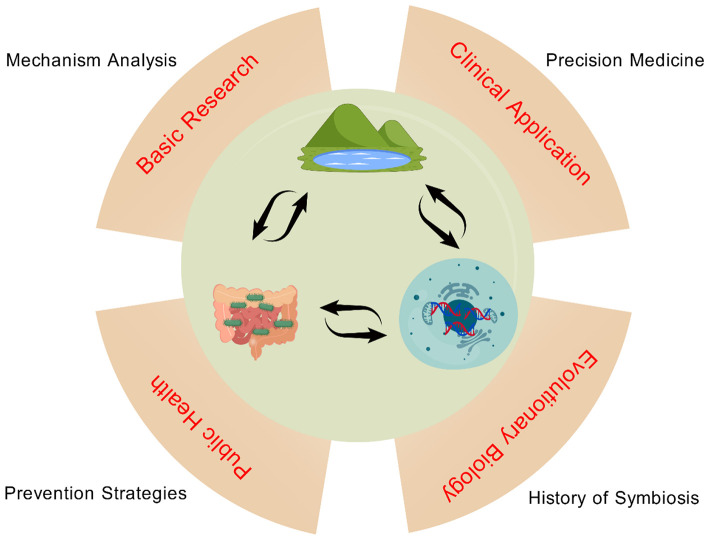
Integrated framework of host-microbiota-environment interactions and translational implications. The central circle depicts bidirectional crosstalk between the gut microbiota **(left)**, host genome/cellular system **(right)**, and environment **(top)**, emphasizing their dynamic, reciprocal relationships. Four translational domains surround this core: Basic Research (Mechanism Analysis): Defines molecular, cellular, and immunological mechanisms of host-microbiota-environment interactions to clarify disease pathogenesis; Clinical Application (Precision Medicine): Translates microbial signatures into diagnostic biomarkers and personalized interventions (e.g., probiotics, prebiotics) for disease management; Evolutionary Biology (Symbiosis History): Explores long-term host-microbiota co-evolution, including effects of human migration and dietary shifts on microbial adaptation; Public Health (Prevention Strategies): Uses population-scale microbiota data to develop chronic disease (e.g., obesity, diabetes) prevention strategies.

In summary, research on the “ethnicity-gut microbiota” axis fundamentally explores the multidimensional interactions among “host genetics, environment, and microorganisms.” Its significance spans fundamental research (mechanism elucidation), clinical applications (precision medicine), public health (prevention strategies), and evolutionary biology (symbiosis history). This research provides a foundation for developing personalized prevention strategies (e.g., dietary interventions) or precision treatment plans (e.g., probiotics targeting specific microbiota/fecal microbiota transplantation) tailored to different ethnic populations, ultimately contributing to improved health outcomes and reduced health disparities worldwide (Figure 6).

## 5 Future research directions for the “Ethnicity-Gut Microbiota” relationship

Future research on ethnicity and gut microbiota will follow a multidimensional, interdisciplinary trajectory, building on current knowledge to address existing gaps and explore new frontiers.

First, large-scale, multiethnic cohort studies are needed to further refine the theoretical foundation of the “Ethnicity-Gut Microbiota” relationship. Existing studies, such as the MetaHIT project, have established cross-ethnic gut microbiome reference gene sets (e.g., a database containing 9.88 million microbial genes), revealing gut microbiome variations across different ethnic groups (Qin et al., 2010). However, these studies have primarily focused on Europe, Asia, and North America, with limited representation from other regions. Further expansion of sample coverage is needed, particularly among non-Western populations such as those in Africa and Latin America, to validate the universality of existing conclusions and identify region-specific patterns of gut microbiota variation. These large-scale studies should also collect comprehensive data on genetic background, lifestyle factors, and health outcomes to enable a more holistic understanding of the factors influencing the “Ethnicity-Gut Microbiota” relationship.

Second, by integrating genome-wide association studies (GWAS) with metagenomic data, we will identify racially specific genetic variants influencing microbial community composition, thereby refining our understanding of the molecular mechanisms underlying racial differences. This integration will allow for the identification of specific genes and genetic pathways that interact with gut microbiota, providing insights into the genetic basis of gut microbial variations among ethnic groups. Furthermore, by examining how gut microbiota and their metabolites differentially affect host epigenetics across racial groups, we will enhance our comprehension of epigenetic regulatory mechanisms.

Third, further elucidate the functional differences of specific bacterial genera across different ethnic groups and refine their correlations with ethnicity-related susceptibility to diseases. This requires not only identifying the presence of specific bacteria but also understanding their metabolic functions and interactions with the host. For instance, research could focus on determining how the metabolic products of specific bacteria differ among ethnic groups and how these differences contribute to variations in disease risk. Clarifying the underlying mechanisms will provide a theoretical foundation for designing precise probiotic or fecal microbiota transplantation (FMT) protocols tailored to different ethnic groups.

Additionally, longitudinal studies can be conducted to construct models of gut microbiota dynamics. By tracking the microbial changes in infants across different ethnic groups from birth through adulthood to old age, we can gain insights into the long-term development of gut microbiota and identify critical periods for intervention. These studies can also explore the feasibility of microbiota rejuvenation strategies—such as transplanting microbiota from younger donors—to improve health outcomes in older individuals.

Finally, the integrated application of multi-omics technologies and machine learning will play a crucial role in advancing “Ethnicity-Gut Microbiota” research. By combining metagenomic, metatranscriptomic, metabolomic, and proteomic data, we can construct ethnicity-specific gut microbiome functional networks that provide a comprehensive view of the interactions between gut microbiota and the host. Machine learning algorithms can then be used to analyze these complex datasets, develop disease risk prediction tools based on ethnicity-specific microbiome characteristics, and identify potential therapeutic targets. For example, support vector machine (SVM) models can integrate clinical data with microbial biomarkers to predict the response of immune thrombocytopenia (ITP) patients to glucocorticoid therapy (Liu et al., 2025). Future work should extend this approach to complex diseases such as diabetes and cancer, while validating the models' generalizability across different ethnic groups.

It is crucial to note that when conducting research involving ethnically diverse populations, ethical considerations must be integrated to ensure studies are carried out in a culturally sensitive way and that the benefits of the research are shared equitably.

## 6 Summary

Research on ethnicity and gut microbiota bridges fundamental science with clinical practice, extending far beyond academic exploration to have profound implications for global health. By decoding the ethnic patterns of microbial diversity, this field is propelling medicine from population-based to personalized approaches, offering a new paradigm for achieving global health equity. The complex interplay between genetics, geography, and lifestyle in shaping gut microbiota among different ethnic groups highlights the need for interdisciplinary collaboration to fully understand the “Ethnicity-Gut Microbiota” relationship.

Future efforts must strengthen collaboration between microbiologists, geneticists, epidemiologists, clinicians, and anthropologists to achieve breakthroughs in mechanism elucidation, technology translation, and ethical governance. Mechanism elucidation will involve uncovering the molecular and cellular pathways through which genetic, geographic, and lifestyle factors interact to shape gut microbiota. Technology translation will focus on developing practical applications such as personalized probiotics, targeted dietary interventions, and microbiome-based diagnostic tools. Ethical governance will ensure that research involving diverse ethnic populations is conducted in an ethical and responsible manner, protecting the rights and interests of participants and promoting equitable access to the benefits of research.

Ultimately, the goal of “Ethnicity-Gut Microbiota” research is to realize precision health management grounded in gut microbiota insights. By understanding the unique gut microbial profiles of different ethnic groups and the factors that shape them, we can develop targeted strategies to prevent and treat diseases, reduce health disparities, and improve the health and wellbeing of individuals worldwide. This research represents a promising frontier in medicine and public health, with the potential to transform our approach to healthcare in the years to come.
